# Detection of sputum cofilin-1 as indicator of malignancy

**DOI:** 10.1590/1414-431X20187138

**Published:** 2018-05-28

**Authors:** M.P. Rangel, L. Antonangelo, M.M.P. Acencio, C.S. Faria, V.K. de Sá, P.S. Leão, C. Farhat, A.T. Fabro, A. Longatto, R.M. Reis, T. Takagaki, V.L. Capelozzi

**Affiliations:** 1 Departmento de Patologia, Faculdade de Medicina, Universidade de São Paulo, São Paulo, SP, Brasil; 2 Laboratório de Investigação Médica (LIM 14), Faculdade de Medicina, Universidade de São Paulo, São Paulo, SP, Brasil; 3 Divisão de Pneumologia, Instituto do Coração (Incor), Faculdade de Medicina, Universidade de São Paulo, São Paulo, SP, Brasil; 4 Departmento de Patologia, Faculdade de Medicina de Ribeirão Preto – Universidade de São Paulo; Ribeirão Prêto, SP, Brasil; 5 Centro de Pesquisa em Oncologia Molecular, Hospital de Câncer de Barretos, Fundação Pio XII, Barretos, SP, Brasil; 6 Life and Health Sciences Research Institute (ICVS), School of Health Sciences, University of Minho, Braga, Portugal; 7 Laboratório de Investigação Médica (LIM 03), Faculdade de Medicina, Universidade de São Paulo, São Paulo, SP, Brasil; 8 Centro Internacional de Pesquisa/CIPE, AC Camargo Cancer Center, São Paulo, SP, Brasil; 9 PT Government Associate Laboratory, Guimarães, Portugal

**Keywords:** Lung cancer, Cofilin-1, Sputum, Liquid-biopsy, Metastases, Biomarker

## Abstract

Cofilin-1 (CFL1), a small protein of 18 kDa, has been studied as a biomarker due to its involvement in tumor cell migration and invasion. Our aim was to evaluate CFL1 as an indicator of malignancy and aggressiveness in sputum samples. CFL1 was analyzed by ELISA immunoassay in the sputum of 73 lung cancer patients, 13 cancer-free patients, and 6 healthy volunteers. Statistical analyses included ANOVA, ROC curves, Spearman correlation, and logistic regression. Sputum CFL1 levels were increased in cancer patients compared to cancer-free patients and volunteers (P<0.05). High expression of sputum CFL1 was correlated to T4 stage (P=0.01) and N stage (P=0.03), tobacco history (P=0.01), and squamous cell carcinoma histologic type (P=0.04). The accuracy of sputum CFL1 in discriminating cancer patients from cancer-free patients and healthy volunteers were 0.78 and 0.69, respectively. CFL1 at a cut-off value of 415.25 pg/mL showed sensitivity/specificity of 0.80/0.70 in differentiating between healthy volunteers and cancer patients. Sputum CFL1 was also able to identify cancer-free patients from patients with lung cancer. The AUC was 0.70 and, at a cut-off point ≥662.63 pg/mL, we obtained 60% sensitivity and 54% specificity. Logistic regression analysis controlled for tobacco history, histologic types, and N stage showed that cancer cell-associated CFL1 was an independent predictor of death. Smoker patients with squamous cell carcinoma, lymph node metastasis and sputum CFL1>1.475 pg/mL showed augmented chance of death, suggesting lung cancer aggressiveness. CFL1 presented diagnostic value in detecting lung cancer and was associated to tumor aggressiveness.

## Introduction

Lung cancer (LC) is the leading cause of tumor death worldwide, and an effective test for its early detection has been an elusive goal for decades. Previous randomized screening trials assessing combinations of chest radiography, sputum cytology, and low-dose helical computed tomography were inconclusive in showing a mortality benefit from screening (1–3). However, the use of this screening strategy has reduced lung cancer-specific mortality by 20% (4); the majority of patients died once metastasis occurred. This may account for deficiencies in accurate diagnosis and risk stratification. Therefore, the identification and validation of novel biomarkers for LC should be considered a priority (5).

In several types of cancer, intracellular and extracellular proteins have shown to be potential diagnostic markers present in blood and secretions, such as saliva, sputum, and urine (6,7). Cofilin-1 (CFL1) is an actin-binding protein that is essential for the depolymerization of actin filaments (8). By inducing CFL1 phosphorylation, Rho abolishes the actin-binding activity of CFL1, thereby enhancing the polymerization of actin filaments. LIM kinase (LIMK) can regulate actin dynamics through the phosphorylation of CFL1 (9). Thus, Rho regulates CFL1 via LIMK, and this signal transduction pathway modulates actin assembly in many cell types in response to various extracellular stimuli, playing a key role in cell migration and cytokinesis (10). CFL1 has been reported to be directly associated to invasion, metastasis, and chemoresistance of various human malignant solid tumors (11–14). Furthermore, it has also been found to be a good tumor biomarker present in the plasma of patients with lung cancer at advanced stages (15). However, no previous studies have considered sputum CFL1 expression as a diagnostic or prognostic marker in LC. The sputum expression of CFL1 and its clinical implication in LC was investigated in the present study.

## Material and Methods

### Patients

This research was carried out in accordance with the Declaration of Helsinki and the study was approved by Universidade de São Paulo Ethics Committee (#256/10). Written informed consent was obtained from all patients.

A cohort of 73 consecutive patients with lung cancer at the Instituto do Cancer and Hospital do Cancer, Barretos, SP, Brazil were included. Moreover, 13 patients classified as cancer-free and 6 as healthy volunteers were included. The cancer-free patients (9 men and 4 women) had a median age of 62 years (range 32–78 years) and presented symptoms of chronic bronchitis. From these patients, 4 were smokers, 2 were non-smokers, 6 were former smokers, and we did not have this information for one patient. Former smokers were defined as patients who had left the tobacco habit for more than 1 year. All 6 healthy volunteers (one man and five women) were selected during the investigation for non-pulmonary diseases. The median age was 73.5 years (range 65–78 years) and 2 patients were smokers.

### Sputum samples

Spontaneous sputum was collected from LC patients just prior to bronchoscopy, and sputum induction through 3 inhalations of 4% hypertonic saline (7 min each) was used for cancer-free patients and healthy volunteers. The sputum samples were stored on ice until processing. To minimize salivary contamination, the samples were visually examined in sterile Petri dishes and then stored at −80°C until the analyses.

### Detection of CFL1 in sputum

The levels of CFL1 were determined by a ‘sandwich’ ELISA test according to the manufacturer's guidelines (USCN Business Co. Ltd, USA). Sputum samples and control standards were added to pre-sensitized plates with anti-CFL1 antibody and incubated at room temperature (RT) for 2 h. After incubation, a biotinylated conjugate antibody was added and incubated at RT for 2 h. Then, streptavidin HRP was added to plates and incubated at RT for 30 min. The plates were washed with Wash Buffer (PBS + Tween 20) 6 times. Revelation was done by adding H_2_O_2_ with tetramethyl benzidine and the reaction was blocked with 30% H_2_SO_4_. The reading was taken in an ELISA reader (Power Wave Bio-Tek, USA) using a 450 nm filter. The minimum detectable level for CFL1 was 78.1 pg/mL. The assays were run in triplicate and the results are reported as means±SD.

### Statistical analysis

Statistical analyses were performed using SPSS Inc. version 18 (USA). Descriptive analysis of quantitative and qualitative data was done, and normal distribution verified with Kolmogorov-Smirnov test. The comparison among histological types, demographics data, and sputum CFL1 was done with Fisher's exact test and chi-squared test. ROC curves were used to evaluate the accuracy of sputum CFL1 in the diagnosis of LC, measured by the area under the curve (AUC). The cut-off values depicted in ROC curves represent the point at which the sensitivity and specificity simultaneously demonstrate higher values (16). Logistic regression was used to evaluate the risk of death for patients with lung cancer based on the levels of CFL1. The Bonferroni method was also used, if necessary. A P value of ≤0.05 was considered to be significant.

## Results

Clinical and pathological characteristics including age, gender, tumor stage, histological types, and tobacco history are reported in Table 1. Ten patients died and 63 were alive until the end of the study.

**Table 1.** Clinical and pathological features of patients with lung cancer.

Patients	73 patients
Age (years)	60 (37–89)^a^
Gender (M/F)	49/24
T stage	
1	11
2	19
3	14
4	29
N stage	
0	10
1	14
2	22
3	27
M stage	
0	33
1	40
Stage	
IB	10
IIA	2
IIB	1
IIIA	5
IIIB	16
IV	39
Histologic types	
SCC	30
AD	33
LCC	5
SCLC	5
Tobacco history	
Yes	18
No	12
Former	43
Follow-up (months)	27 (0–70)^a^

Data are reported as median (range)^a^ and numbers. SCC: squamous cell carcinoma; AD: adenocarcinoma; LCC: large cell carcinoma; SCLC: small cell lung carcinoma.

**Figure 1.**
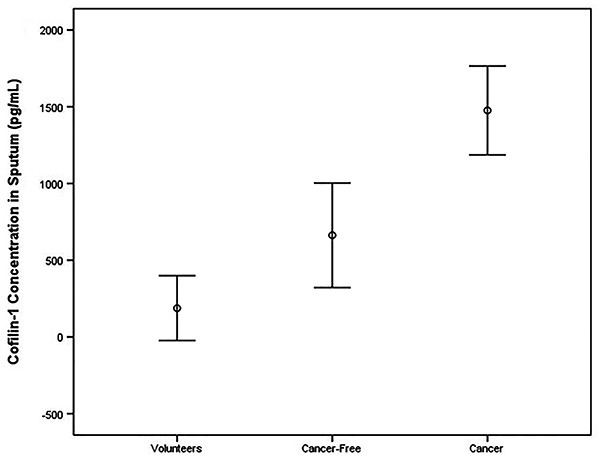
Levels of sputum cofilin-1 in healthy volunteers, cancer-free and lung cancer patients. Data are reported as means±SD. P=0.01 for between groups comparisons (chi-squared test).

**Figure 2.**
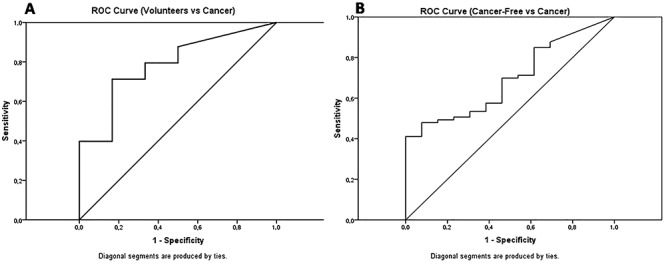
ROC curves representing the diagnostic ability of cofilin-1 level in sputum (*A*) to distinguish cancer patients from healthy people (AUC=0.78) and (*B*) to differentiate cancer-free patients from cancer patients at high risk (AUC= 0.69).

**Figure 3.**
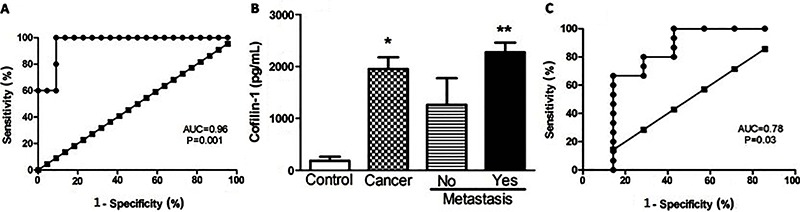
Analysis of cofilin-1 levels in patients with >25 pack-year smoking frequency. *A*, ROC curve for the discrimination between normal controls and patients with lung cancer. *B*, Cofilin-1 in patients with metastasis. *C*, ROC curve for the discrimination between presence or absence of metastasis based on cofilin-1 levels. In *B*, data are reported as means±SD. *P<0.05 compared to control; **P<0.05 compared to no metastasis (chi-squared test).

The results of CFL1 stratified by the presence or absence of LC are shown in Table 2. A significant increase of sputum CFL1 was observed in patients with lung cancer (1475.83±145.35 pg/mL) compared to cancer-free patients (662.63±5.74 pg/mL) and healthy volunteers (415.25±3.68 pg/mL) (P=0.01) (Figure 1). ROC curves constructed to demonstrate the accuracy of sputum CFL1 in discriminating cancer patients from cancer-free patients and healthy volunteers were 0.78 and 0.69, respectively. At a cut-off point of CFL1≥415.25 pg/mL, the sensitivity and specificity was 70 and 80% (Figure 2A). Sputum CFL1 was also able to distinguish cancer-free patients from patients with LC. With the AUC at 0.70 and the cut-off point ≥662.63 pg/mL, we obtained 60% sensitivity and 54% specificity (Figure 2B). In a clinical setting, with a >25 pack-year smoking history, CFL1 could be useful to predict lymph node metastasis with an AUC of 0.96 (Figure 3).

**Table 2.** Descriptive analysis of cofilin-1 in the sputum of cancer patients, cancer-free patients, and volunteers.

Cofilin-1	n	Mean (pg/mL)	Standard deviation	Standard error	95% Confidence Interval for mean		Minimum	Maximum
					Lower bound	Upper bound		
Volunteers	6	415.25	578.65	236.23	−192.00	1022.50	78.10	1554.80
Cancer-free	13	662.63	564.01	156.43	321.79	1003.46	78.10	1521.70
Cancer	73	1475.83	1241.87	145.35	1186.07	1765.58	78.10	4239.40
Total	92	1291.75	1189.52	124.01	1045.41	1538.09	78.10	4239.40

The univariate analysis showed that a high concentration of sputum CFL1 is correlated to T4 stage (P=0.01), presence of lymph node metastases (P=0.03), tobacco history (P=0.01), and squamous cell carcinoma subtype (P=0.04). Therefore, we constructed logistic regression models with these variables to determine the risk of distant metastases; the best model is shown in Table 3. This model, controlled for smoking history, histological subtypes, and N staging, showed that sputum CFL1 of patients with LC is an independent predictive factor of death [OR=5.77 (0.78–42.74)] (-2Log likelihood d=41.32, P<0.01). On the other hand, squamous cell subtype with sputum CFL1>1.475 pg/mL showed an augmented chance of death, reinforcing the role of CFL1 as a potential biomarker of aggressiveness in LC.

**Table 3.** Logistic regression model assessing independent predictors of death.

Variables	β coefficient	Standard error	Wald	P value	Exp (β)	95% CI for odds ratio	
						Lower	Upper
Tobacco history			5.47				
Negative	−2.84	1.24	5.27	0.02	0.06	0.005	0.66
N stage			4.48				
N_0_	−3.79	0.93	5.18	0.02	0.08	0.006	0.78
Histologic type			3.39				
Squamous cell carcinoma	1.75	1.02	2.95	0.05	4.57	0.45	32.67
Cofilin-1							
>1.475 pg/mL	1.75	0.01	3.93	0.01	5.77	0.78	42.74

−2 Log likelihood = 41.32; P=0.001. Guimarães, Portugal. N_0_: absence of lymph node metastasis.

The univariate analysis showed that a high concentration of sputum CFL1 is correlated to T4 stage (P=0.01), presence of lymph node metastases (P=0.03), tobacco history (P=0.01), and squamous cell carcinoma subtype (P=0.04). Therefore, we constructed logistic regression models with these variables to determine the risk of distant metastases; the best model is shown in Table 3. This model, controlled for smoking history, histological subtypes, and N staging, showed that sputum CFL1 of patients with LC is an independent predictive factor of death [OR=5.77 (0.78–42.74)] (-2Log likelihood d=41.32, P<0.01). On the other hand, squamous cell subtype with sputum CFL1>1.475 pg/mL showed an augmented chance of death, reinforcing the role of CFL1 as a potential biomarker of aggressiveness in LC.

## Discussion

We demonstrated that patients with LC present significant increases in CFL1 levels in sputum, emerging therefore as a potential biomarker for LC diagnosis. In addition, we found that cancer patients contain higher concentrations of sputum CFL1 compared to cancer-free patients and healthy volunteers. This finding is relevant since it represents an unprecedented biomarker to be used for LC screening, which does not require invasive procedures. Although induced sputum collection is a slightly less comfortable method for the patient than spontaneous sputum collection, adverse effects are minimal, and usually associated with bronchoconstriction. The present study did not show any adverse effects during or after the procedure.

The relevance of sputum CFL1 remained high even when the concentration was compared between cancer patients and healthy volunteers, cancer patients and cancer-free patients, and cancer-free patients and healthy volunteers. As far as we know, such molecules have never been assayed in the sputum; however, our results corroborated those by Zheng et al. (15).

We also found an association between the concentration of CFL1 and squamous cell carcinoma subtype. In a previous study, our group showed that hyaluronic acid, a GAG not sulfated component of the extracellular matrix, was increased in sputum of patients with LC (7) particularly in squamous cell carcinoma, and probably associated to the more central location of this subtype of cancer in the respiratory tree. In addition, other tests performed in sputum, as cytology, are more useful in cases of squamous cell carcinoma, since its more central location favors a higher production of sputum (17).

In our study, a concentration of CFL1 above 468 pg/mL demonstrated sensitivity and specificity of 80 and 70%, respectively, in discriminating patients with lung cancer from healthy volunteers. The disadvantage of this cut-off point is that around 30% of patients with cancer can have a false negative diagnosis making the test inconclusive, and requiring conventional methods for diagnostic elucidation, such as bronchoscopy. However, the results presented in this study are highly relevant, since the search for diagnostic markers related to LC has been the subject of much research in recent decades. According to Gadgeel (17), various study groups have been trying to find a serum or sputum biomarker for lung cancer detection, but to date, none of these markers showed broad application in clinical practice. Most of the conventional techniques are invasive and cause great discomfort and anxiety, or even bring painful side effects to the patients (17,18). Therefore, adjuvant screening tests would be highly beneficial for patients with a medical history of or high risk for developing lung cancer.

The levels of CFL1 in sputum seem to present an impact on the natural history of patients with lung cancer. Interestingly, we showed that high a concentration of CFL1 correlated to T4 stage, lymph node metastases, and tobacco history. In fact, by analyzing the logistic regression controlled for tobacco history, histological types, and staging, we observed that the concentration of sputum CFL1 was an independent predictor of risk of lymph node metastases in patients with lung cancer. These results indicate that the concentration of this protein in sputum is related to tumor progression, probably as a consequence of modifications in the extracellular matrix (ECM) earlier in the carcinogenesis process, i.e., at the stage of neoplastic transformations. According to Wistuba et al. (19), this is a phase of ECM tissue remodeling, which can affect sputum composition and production of its components. As mentioned earlier, the interaction between tumor cells and the neoplastic microenvironment, represented by the ECM, influences the invasive and metastatic properties of tumors.

Despite the difference in CFL1 behavior among the studied groups, some limitations of the study should be highlighted. First, despite the small number of volunteers included in the control group, we have to consider the difficulty of recruiting healthy people with the same age of patients with lung cancer for collecting induced sputum samples. Because of this difficulty, there is a difference in the number of patient cases and the number of normal cases. However, the considerable increase in CFL1 in the sputum of patients with lung cancer suggest that changes have already occurred at the beginning of carcinogenesis, giving this protein a promising role as a lung cancer biomarker.

Another limitation of the study is that samples were stored at -80°C, hindering a cytological study of sputum, which would certainly add sensitivity to the diagnosis of lung cancer.

In addition, it should be considered that at the time of sputum collection, some patients were recently diagnosed with lung cancer, thus had a short period of follow-up to be evaluated.

Considering the results presented here, CFL1 in sputum could be considered a promising screening test for application in clinical practice, since it is an alternative tool to detect the disease by means of a non-invasive procedure. However, for its application in the diagnostic routine, a larger number of cancer cases should be studied. Nevertheless, the results observed and discussed here indicate an unquestionable role of this protein in the natural history of lung cancer.
